# Integrating CVH and LVH metrics into an optimization strategy for the selection of Iris collimator for Cyberknife Xsight lung tracking treatment

**DOI:** 10.1002/acm2.13136

**Published:** 2021-01-11

**Authors:** Feng Xiao, Yu Chang, Sheng Zhang, Zhiyong Yang

**Affiliations:** ^1^ Medical Physics School of Physics and Technology Wuhan University Wuhan 430072 China; ^2^ Cancer Center Union Hospital Tongji Medical College Huazhong University of Science and Technology Wuhan 430022 China

**Keywords:** CyberKnife, lung stereotactic radiotherapy, normal tissue sparing, target coverage

## Abstract

**Purpose:**

We conducted this study to construct a target coverage‐volume histogram (CVH) and leakage‐volume histogram (LVH) metrics and optimization strategy for the selection of the Iris collimator in Cyberknife Xsight lung tracking treatment through a retrospective analysis of target structures and clinical data.

**Methods and Materials:**

CVH and LVH metrics were retrospectively analyzed for 37 lung cancer patients. CVH and LVH were the same as dose‐volume histogram (DVH), but with a coverage and leakage replacing dose. For each patient, Iris collimator was optimized and selected based on CVH and LVH metrics. The CVH and LVH metrics were then compared to ascertain differences in 95% (C95) or 90% (C90) of the target coverage thresholds. The planning target volume (PTV) C95 and C90 coverage, absolute mean leakage value, leakage/coverage ratio, selected collimator diameter (Φ), Φ/length of the long axis of PTV (A_max_), and Φ/length of the short axis (A_min_) of PTV were compared. The correlation of the absolute mean leakage value, leakage/coverage ratio, Φ/A_min_ and Φ/A_max_ were evaluated.

**Results:**

For each patient, the PTV C95 coverage (70.45 vs 63.19) and C90 coverage (77.25 vs 69.96) were higher in the C95 coverage threshold group compared to the C90 coverage threshold group. The leakage/coverage ratio (0.56 vs 0.69) and absolute mean leakage value (0.56 vs 0.61) were lower in C90 coverage threshold group than in C95 coverage threshold group. The Spearmen correlation test showed the Φ/A_min_ were significantly correlated with leakage/coverage ratio and absolute mean leakage value. Upon analysis of the selected collimator diameters, the mean value of Φ/A_min_ of the optimized collimator diameters was found to be 1.10.

**Conclusion:**

The CVH and LVH analysis is able to quantitatively evaluate the tradeoff between target coverage and normal tissue sparing.

## 1 INTRODUCTION

The CyberKnife Robotic Radiosurgery System (CKS, Accuray, Inc., Sunnyvale, CA, USA) has been widely used for stereotactic body radiation therapy (SBRT).^1^, ^2^, ^3^ For lung and liver SBRT, the therapeutic ratio is essential to ensure adequate coverage of moving targets while sparing the surrounding normal tissues. The amount of irradiated volume in CyberKnife treatment depends on applied motion management strategy, adopted safety margins, and beam collimator selection.^4^, ^5^, ^6^ Most of the CyberKnife treatment retrospective studies have focused on improving tracking accuracy and reducing the planning target volume (PTV) margins.^1^, ^7^, ^8^, ^9^, ^10^, ^11^ Some studies have investigated the relationship between dose uncertainty of CyberKnife Xsight Lung Tracking (XLT) treatment and the collimator diameters of XLT plan, and have shown that the intrafractional dose uncertainty can be reduced with large collimators.^12^, ^13^ However, the XLT plans with large collimators will certainly irradiate more surrounding normal tissues.^5^, ^14^, ^15^ Hence, the selection of collimator diameters of XLT plans is a tradeoff between the target coverage and the normal tissue sparing.

The CyberKnife VSI system at our institute has an Iris variable aperture collimator (collimator). This collimator allows 12‐field diameters to be used without a manual exchange of collimators.^16^ At our institution, in order to improve the plan quality and time efficiency, one collimator is used for small tumors in CKS treatment and two collimator combinations (small and large) are used for the large tumors in CKS treatment.^4^, ^6^, ^17^ We previously analyzed the intrafractional dose uncertainty in the CyberKnife XLT treatment, and found that the plan robustness is better with large collimator.^12^ However, the relevance between the proposed value of the collimator diameter in the CyberKnife XLT treatment plan and the intrafractional target tracking error has not yet been investigated. In addition, the appropriate value of the collimator diameter that can retain plan robustness and not sacrifice too much surrounding lung tissues is still unclear.

Therefore, we performed a simulation based on quantitative evaluation of target coverage and normal tissue sparing as a function of collimator diameter selected in CyberKnife treatment. We pursued this aim through a retrospective analysis on the geometry of target structures from the plans and treatment data extracted from the log files.

## 2 MATERIAL AND METHODS

### 2.A Patient data

Overall, 37 patients with lung cancer that were treated with the Cyberknife VSI system between April 2018 and April 2019 were included in this study. All patients were enrolled through an institutional review board‐approved retrospective data collection protocol. Patients were immobilized with vacuum pads in a supine position, with their arms along their sides. The gross tumor volume (GTV) was contoured on the exhale phase CT images and used for target tracking using the XLT system.^18^ The GTV to clinical target volume (CTV) margin for inclusion of microscopic extension of the tumor was 2 mm. Depending on the specific clinical scenario and a previous study, the PTV was derived using a 4 mm expansion from CTV in all three directions in order to account for treatment uncertainties and residual errors at our institute.^1^ Patients were planned using XLT system with a nonisocentric method in the Multiplan treatment planning system. The plan was optimized using sequential optimization algorithm with delivery of 30–40 Gy in 3‐7 fractions. Patient and target characters are shown in Table 1. The collimator in Cyberknife VSI system allows 12 field diameters to be used, including 5, 7.5, 10, 12.5, 15, 20, 25, 30, 35, 40, 50, and 60 mm that are defined at 800 mm from the focal spot.^16^

**TABLE 1:** Patient and target motion characteristics (Patient number = 37).

Characteristics	
Tumor location	
Upper lobe	3
Middle lobe	21
Lower lobe	13
Fractions: Mean ± SD	5.11 ± 1.73
Treatment time: Mean ± SD (minute)	30.03 ± 6.12
PTV volume: Mean ± SD (cm^3^)	41.15 ± 30.17
PTV long axis length (mm)	56.80 ± 17.26
PTV short axis length (mm)	36.36 ± 10.44
Treatment delivery error*: Mean ± SD (mm)	
SI direction	0.86 ± 0.52
LR direction	1.21 ± 0.81
AP direction	0.58 ± 0.31

* Treatment delivery error is combined with correlation error and prediction error.

### 2.B Treatment delivery error

The main intrafraction error sources in CyberKnife XLT treatment are segmentation and deformation errors, both of which are associated with segmentation and deformation of the moving tumors in specific patients. Additionally, correlation and prediction errors are associated with the accuracy of the correlation and predictive models in XLT system.^1^ The CyberKnife XLT treatment correlation error is defined as the difference between the collimator tracking position and the target position, which is measured through imaging, since calculating the tumor position from x‐ray images is the gold standard in order to locate tumor position.^1^, ^7^ The CyberKnife XLT treatment prediction error is derived by comparing the output tumor position of the predictive model with the correlation model at 115 ms in the future.^1^, ^7^ The correlation and prediction errors are the common uncertainties associated with treatment delivery of the XLT system, as analyzed in the previous studies.^8^, ^9^, ^10^, ^19^, ^20^ The framework of the correlation and prediction error calculation was performed as detailed in our previous study.^1^ Owing to a lack of volumetric images of targets during treatment, the segmentation and deformation errors were difficult to calculate in this study. The target motion is considered to be rigid during treatment. We only analyzed uncertainties of correlation and prediction models in this study.

### 2.C CVH and LVH analysis

**FIG. 1:**
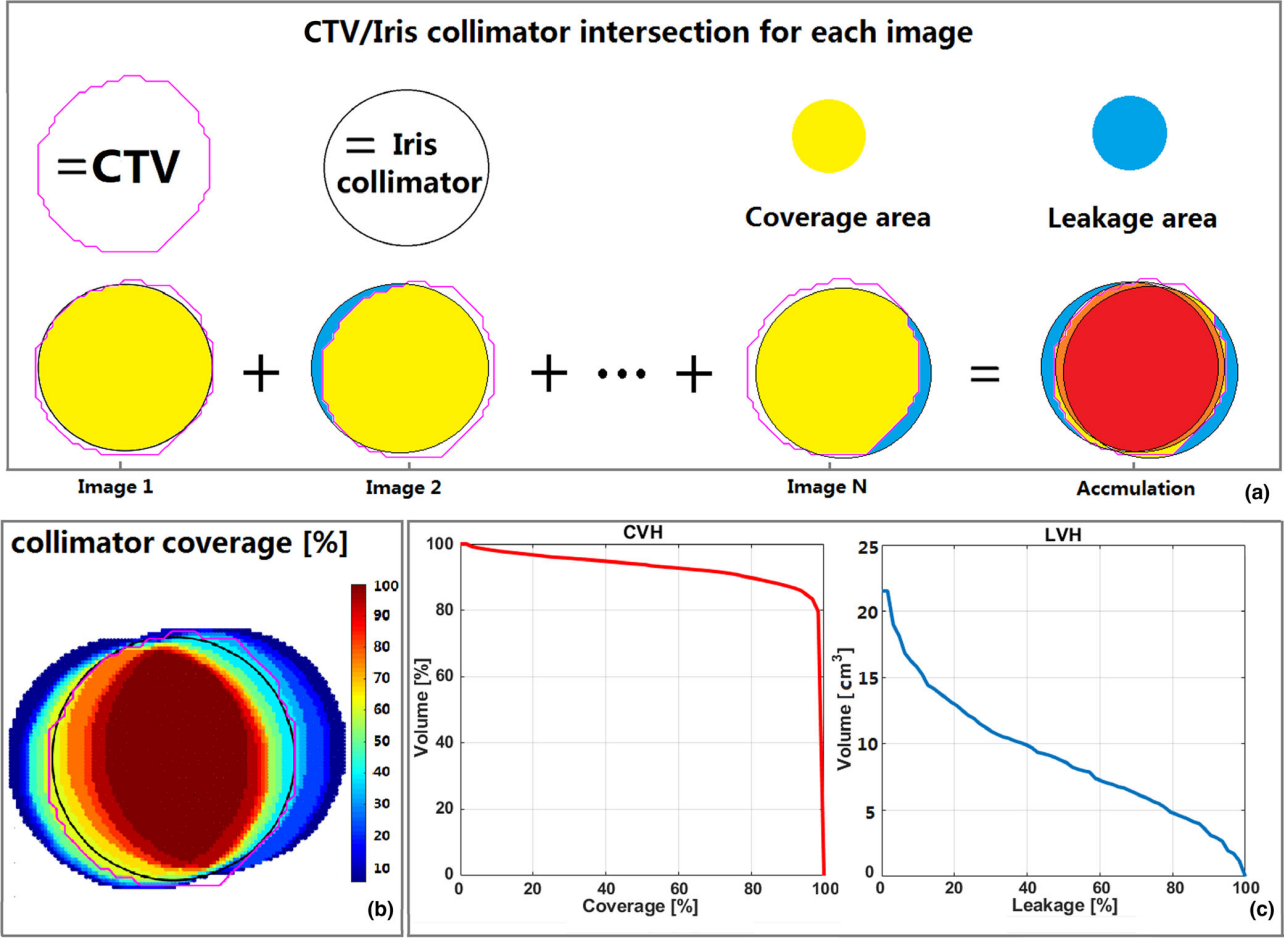
Exemplification of the CTV coverage and normal tissue leakage calculation. Panel a: for each control image acquired during treatment (1, 2,...,N), the intersection (yellow area) between the CTV (pink structure) and the projection of the collimator (black circle) is calculated and accumulated as CTV coverage; the area (blue area) which is not in the projection of the collimator (black circle) but not included in the CTV (pink structure) is calculated and accumulated as normal tissue leakage. Panel b: the coverage map of the voxels with the percentage of the accumulated x‐ray images coverage. Panel c: cumulative coverage‐volume histogram (CVH) and leakage‐volume histogram (LVH). In this exemplification, about 90% of the PTV volume receives 90% coverage (C90 is 90%); about 5 cm^3^ of normal tissues receives 80% leakage.

The coverage‐volume histogram (CVH) and leakage‐volume histogram (LVH) were generated to provide a quantitative evaluation of the tradeoff between sparing normal tissues and the target coverage based on the simulation of delivery correlation and prediction uncertainties. We simulated the beam delivery process as follow. First, we calculated the "real" field size of the isocentric and nonisocentric beams at the center of the target in the order of the x‐ray imaging time points and then aligned them to the points at the relative movement vector of the target center, which was obtained through the ModelPoint.log. The intersection of target volume and irradiated volume was then calculated in correspondence to the intersection between CTV, which was centered on the real‐time x‐ray imaged target position (serving as ground truth) and the collimator, which was centered on the predicted target position (a ball with the collimator in diameter centered on the predicted target position) [Fig. 1(a)]. In this way, we defined the target coverage of each voxel in the target volume as the possibility of such voxel being included in the intersection volume in the x‐ray images in total fractions [Fig. 1b, 1c]. The leakage volume was calculated as voxels in the collimator aperture centered on the predicted target position, but not included in CTV, which was centered on real‐time x‐ray imaged target position (Fig. 1a). The leakage volume refers to the normal tissues around the tumor which were irradiated in collimator aperture during treatment. We defined the leakage possibility of absolute leakage volume as a possibility of such voxel not being included in the intersection volume of x‐ray images in total fractions (Fig 1b, c).

Similar to cumulative dose‐volume histograms, **CVH** i**s derived from the cumulative target coverage‐volumetric histogram**, **while LVH is derived from the cumulative leakage possibility‐ absolute volumetric histogram**. The coverage of the 95% or 90% of CTV in total fractions (C95 or C90 CTV) and the area under coverage curve was chosen as CVH evaluation metrics (Fig. 1c). CVH, C95 and C90 values were calculated for all patients. We considered the coverage rate of 95% and 90% of CTV volume greater than 90% (C95/C90 of CTV ≥ 90%) as the two thresholds in order to determine whether the target coverage was accomplished.^7^, ^21^, ^22^ The area under the leakage curve and absolute mean leakage value (the value of the area under leakage curve divided by the leakage volume) were calculated as LVH evaluation metrics (Fig 1c). The leakage/coverage ratio (the value of the area under leakage curve divided by the area under coverage curve of PTV) was also calculated to evaluate the degree of compromise between the sparing normal tissues and collimator coverage.

### 2.D Collimator diameter optimization

**FIG. 2:**
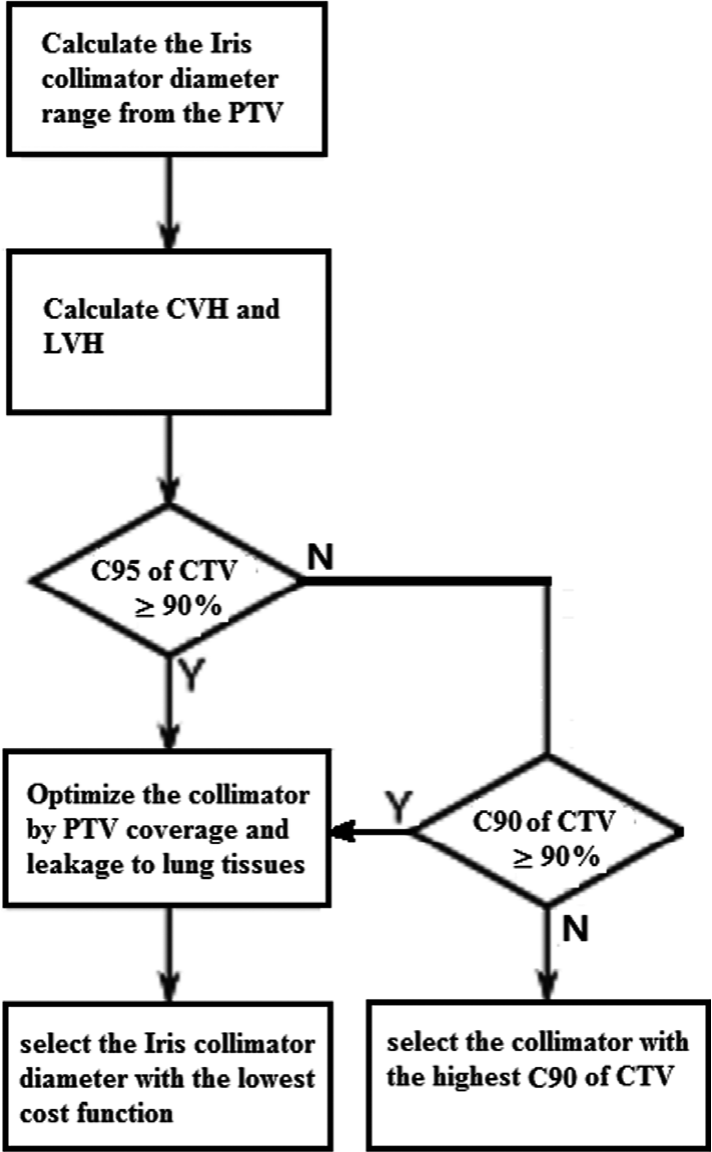
Flow chart of optimization strategy for Iris collimator selection.

We adopted the CVH and LVH analysis strategy to apply an a‐posteriori collimator diameter optimization on a patient‐specific basis. The CVH and LVH optimization program was performed using MATLAB^®^ (MathWorks, Natick, MA). A flow chart of the optimal strategy is depicted in Fig. 2, and is depicted in more detail below.

Step 1: The lengths of the long and short axes of PTV (A_max_ and A_min_) for each patient were first calculated to evaluate the possible ranges of the collimator diameter for the specific patient. According to the previous study, the most suitable collimator diameter was selected from [0.5⋅A_min_, A_max_ + 5mm].^6^ The collimator diameter below the 0.5⋅A_min_ is too small, not suitable for selection. The collimator diameter above the value of the long axes of the target is too big for selection, since the surrounding normal tissues will be irradiated too much.

For step 2, the CVH and LVH of all collimator diameters were calculated and restored for each patient. The collimator diameters which satisfied the C95 of CTV ≥ 90% were initially selected to keep target coverage. If the C95 coverage threshold could not be met, then the collimator diameters which satisfied C90 of CTV ≥ 90% were selected.

For step 3, after selecting out collimator diameters which satisfied target coverage, we set an optimization model to optimize and select the most suitable collimator diameter. The model was based on the C95 of PTV, as well as mean leakage value. The cost function of the optimization model is:

$$F\left(d_{i}\right)=w\cdot C95_{\mathrm{PTV}}\left(d_{i}\right)‐\bar{D_{\mathrm{Leakage}}\left(d_{i}\right)}/V_{\mathrm{alung}}$$

where $d_{i}$ is the value of the collimator diameter I, which is from [0.5⋅A_min_, A_max_ + 5 mm] and $d_{i}$ must satisfy that C95(or C90) of CTV ≥ 90%. Additionally, w is the weight of coverage of 95% of PTV in the plan evaluation, and $V_{\mathrm{alung}}$ is the volume of the affected lung. As mentioned in step 2, the coverage of CTV was satisfied. We set w to zero since the coverage of PTV was not necessary in our institution. For each patient, the most suitable collimator diameter ($d_{i}$) is selected according to the rule that $d_{i}$ has the lowest cost function value $F\left(d_{i}\right)$ of the optimization model.

### 2.E Statistics analyses

SPSS 23.0 software (IBM, Armonk, NY) was used for statistical analyses of all CVH and LVH metrics. In order to evaluate the differences between target coverage and normal tissue sparing obtained from two coverage thresholds before the collimator diameter optimization process; we conducted a paired, two‐tailed Wilcoxon signed‐rank test in order to compare CVH and LVH metrics of C90 and C95 coverage thresholds.

After collimator diameter optimization, we tested the relevance between the selected collimator diameter and LVH metrics. Since the selected collimator diameter is related to PTV volume and axis length, we settled the value of collimator diameter (Φ) divided by the short and long axis lengths of PTV (Φ/A_min_, Φ/A_max_) as an index to determine the relevance between collimator diameters and CVH/LVH metrics while exclude the impact of the PTV volume. We calculated the leakage/coverage ratio, as well as the absolute mean leakage value. We conducted a two‐tailed Spearmen correlation test to evaluate relevance between Φ/A_min_, Φ/A_max_ and the leakage/coverage ratio, absolute mean leakage value. *P* < 0.05 were considered statistically significant.

## 3 RESULTS

The PTV volume (mean ± standard deviation [SD]) was 41.15 ± 30.17 cm^3^, and the length of the long and short axis (mean ± SD) were 56.80 ± 17.26 and 36.36 ± 10.44 cm, respectively.

The correlation errors of all patients were 1.21 ± 0.81 mm, 0.58 ± 0.31 mm, and 0.86 ± 0.52 mm in the left‐right (LR), anterior‐posterior (AP), and superior‐inferior (SI) direction, respectively. The total correlation error (mean ± SD) was 0.88 ± 0.41 mm.

### 3.A Coverage and leakage analysis

**FIG. 3:**
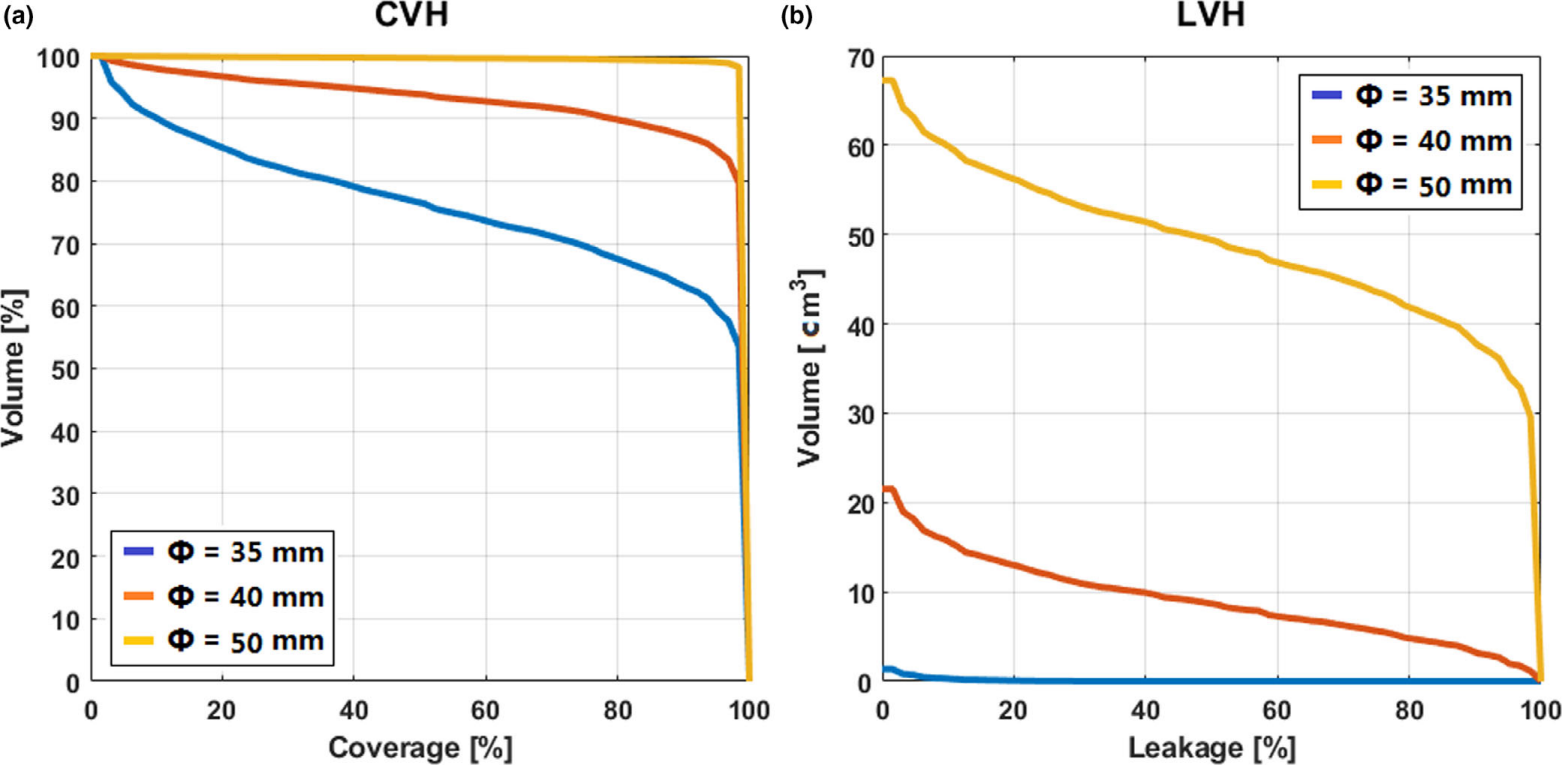
Converge‐volume histograms (CVH) and leakage‐volume histograms (LVH). For the representation case, the CVH (Panel a) and LVH (Panel b) with collimator diameter 35 mm, 40 mm and 45 mm are represented in blue, red, and orange, respectively.

In order to illustrate the differences in dose distribution between the plans, we present the patient in Fig. 1 as a representative case. This patient's CVH and LVH evaluation metrics were obtained for different collimator diameters (Fig. 3). As shown, the larger collimator diameter, such as Φ = 50 mm (the orange line in panel a), the C90 and C95 were higher in CVHs compared to Φ = 35 mm (the blue line in panel a). While the absolute mean leakage value is also larger in LVHs compared to Φ = 35 mm (the blue line in panel b).

The CVH and LVH evaluation metrics of the collimator diameters are summarized in Table 2. The C95 and C90 were two thresholds of target coverage for the selection of collimator diameters prior to the optimization process. The C95 (70.45 ± 16.75 vs 63.19 ± 19.17, *P* = 0.002) and C90 (77.25 ± 13.90 vs 69.96 ± 16.72, *P* = 0.002) of PTV were higher in the C95 coverage threshold group compared to the C90 coverage threshold group. The leakage/coverage ratio (0.56 ± 0.60 vs 0.69 ± 0.61, *P* = 0.002) and absolute mean leakage value (0.56 ± 0.21 vs 0.61 ± 0.20, *P* = 0.002) were lower in the C90 coverage threshold group compared to the C95 coverage threshold group. The selected collimator diameter (38.7 ± 12.1 vs 40.0 ± 12.1, *P* = 0.001), Φ/A_min_ (1.01 ± 0.19 vs 1.09 ± 0.22, *P* = 0.002) and Φ/A_max_ (0.63 ± 0.11 vs 0.70 ± 0.11, *P* = 0.002) is less than in the C90 coverage threshold group compared to the C95 coverage threshold group.

**TABLE 2:** The CVH and LVH evaluation metrics of the collimator diameters are divided into two groups as selected by C95 or C90 thresholds.

Parameter	PTV C90 coverage (%)	PTV C95 coverage (%)	Absolute mean leakage value	Leakage/coverage ratio	Selected collimator diameter	Φ/Amin	Φ/A_max_
C90 CTV group	69.96 ± 16.72	63.19 ± 19.17	0.56 ± 0.21	0.56 ± 0.60	38.7 ± 12.1	1.01 ± 0.19	0.63 ± 0.10
C95 CTV group	77.25 ± 13.90	70.45 ± 16.75	0.61 ± 0.20	0.69 ± 0.61	40.0 ± 12.1	1.10 ± 0.22	0.70 ± 0.11
*P* value	0.002^*^	0.002^*^	0.002^*^	0.002^*^	0.001^*^	0.002^*^	0.002^*^

Abbreviations: ^*^means *P* < 0.05 absolute mean leakage value = the value of the area under leakage curve divided by the leakage volume; C90 = coverage of the 90% of target volume; C95 = coverage of the 95% of target volume; Φ/A_max_ = the value of collimator diameter divided by the long axis lengths of PTV; Φ/A_min_ = the value of collimator diameter divided by the short axis lengths of PTV; The leakage/coverage ratio = the value of the area under leakage curve divided by the area under coverage curve of PTV.

**FIG. 4:**
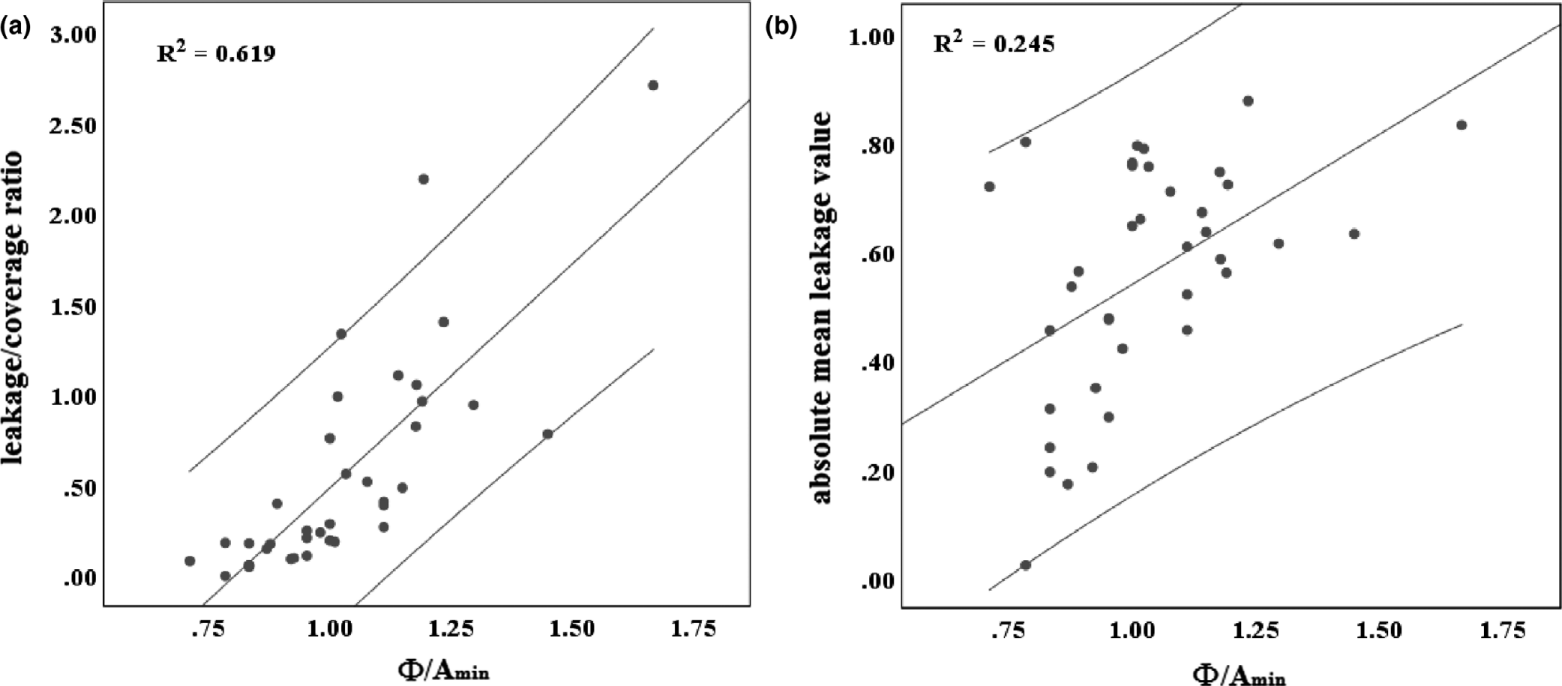
The correlation of leakage/coverage ratio and Φ/A_min_ (Panel a), and the correlation of absolute mean leakage value and Φ/A_min_ (Panel b) for all cases.

The Spearmen correlation test indicated that the optimized collimator diameters index Φ/A_min_ were significantly correlated with the leakage/coverage ratio (R = 0.787, *P* < 0.001) and absolute mean leakage value (R = 0.495, *P* = 0.002). However, no uniform trend of Φ/A_max_ and leakage/coverage ratio (R = 0.232, *P* = 0.166) and absolute mean leakage value (R = 0.092, *P* = 0.590) was found in our study. The relevance between Φ/A_min_ and leakage of surrounding normal lung tissues are shown in Fig. 4.

### 3.B Collimator diameter optimization

**FIG. 5:**
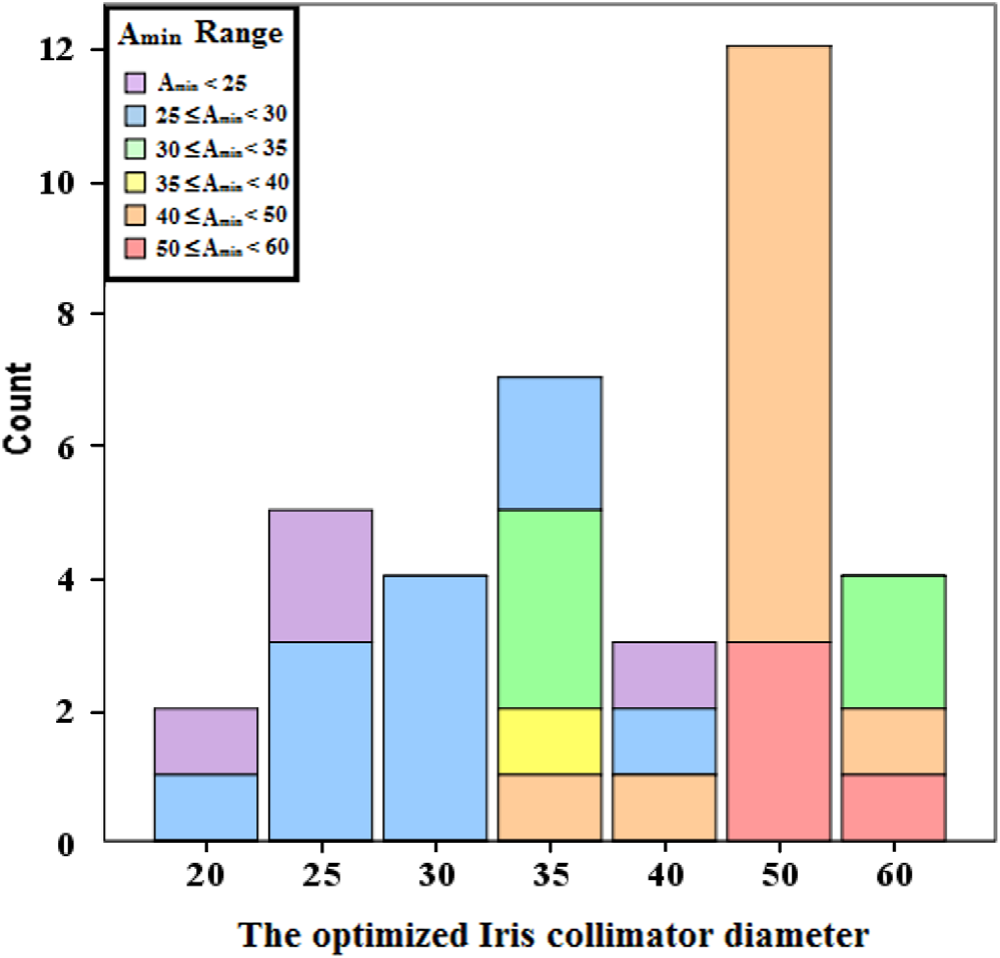
The optimized collimator diameter distributions are showed by different diameters (within different bars) and A_min_ ranges (within different colors).

The collimator diameter optimization results are reported in Table 3. In the A_min_ < 35 mm scenarios, most of the optimized collimator diameters are below A_min_. However, in the A_min_ > 35 mm but < 50 mm scenarios, the distribution of the optimized collimator diameters is slightly wide. Finally, in the A_min_ > 50 mm scenarios, the optimized collimator diameters are almost equal to 50 mm. The mean value of Φ/A_min_ of the optimized collimator diameters is 1.10 ± 0.22 (Table 2). Fig. 5 depicts the optimized Φ distributions grouped by different diameters and A_min_ ranges.

**TABLE 3:** The distribution of the optimized collimator diameters in different A_min_ Ranges.

A_min_ Ranges	The optimized collimator diameters (mm)							Total
	20	25	30	35	40	50	60	
A_min_ < 25	1	2	0	0	1	0	0	4
25 ≤ A_min_ < 30	1	3	4	2	1	0	0	11
30 ≤ A_min_ < 35	0	0	0	3	0	0	2	5
35 ≤ A_min_ < 40	0	0	0	1	0	0	0	1
40 ≤ A_min_ < 50	0	0	0	1	1	9	1	12
50 ≤ A_min_ < 60	0	0	0	0	0	3	1	4
Total	2	5	4	7	3	12	4	37

## 4 DISCUSSION

This study demonstrated the feasibility of applying CVH and LVH evaluation metrics and optimization strategy to select a proposed Φ for CyberKnife treatment. Our results demonstrate that the proposal collimator diameter is 1.1 times that of the short axis length of the target volume in a majority of cases. With this proposal collimator diameter, less normal tissues will be compromised for target coverage.

Several previous studies have investigated the relevance between collimator diameter and the target coverage. Chan *et al*. have found that large collimator is able to reduce dose uncertainty and improve target coverage.^13^ Iwata *et al*. have found that using a smaller collimator (1/2 of the length of a tumor long axis) could lead to a decrease in dose uncertainty.^6^ Ricotti *et al*. applied CVH analysis on PTV and optimized PTV margin on XLT treatment.^23^ Our previous study also applied a dynamic thorax phantom and EBT3 films to evaluate dose uncertainty, and found that the larger collimator can improve target coverage.^12^ In this study, our results were in agreement with these previous studies. Our results indicated that for each patient, the target coverage is higher when a larger collimator diameter is selected, according to a CVH metrics analysis (Fig. 3).

However, normal tissue sparing is also an important factor in selecting for collimator diameter before treatment plan optimization.^24^ We relied on LVH as a metric in order to quantify normal tissue sparing. LVH demonstrates the volume of irradiated normal tissues and possibility of irradiated dose to these normal tissues. In this manner, the target coverage (i.e., PTV coverage) and absolute mean leakage value can be considered together and are effectively related to the adopted collimator diameter. Moreover, we calculated the differences of normal tissue sparing and PTV coverage between strict (C95) and normal (C90) target coverage thresholds. The result shows the trend that in order to keep a higher CTV coverage, the selected collimator diameter of the C95 threshold should be higher than that of the C90 threshold, while the leakage/coverage ratio should be higher in the C95 threshold compared to the C90 threshold.

We focused on using the geometric distances of the mass centers between the targets and collimators to calculate CVH and LVH metrics. First, we established the index (such as Φ/A_min_ and Φ/A_max_) to evaluate the relevance between geometries of target structures and collimators. The Φ/A_min_ is significantly correlated with the LVH metrics (leakage/coverage ratio and absolute mean leakage value) (Fig. 4). This suggests that, excluding the influence of target volume and lengths, a larger relative collimator diameter will irradiate more normal tissues. The reason is that the collimator will miss centered by the correlation error, and more surrounding normal tissues will be included with a larger collimator.

The tradeoff between target coverage and normal tissue sparing depends on the target geometry, as well as the location relationship with the surrounding normal tissues and treatment delivery error.^14^, ^24^ The target geometry and location relationship with the surrounding normal tissues are total patient‐specific factors. These factors could be only quantified using CVH and LVH analysis and optimization. The treatment delivery error is also a patient‐specific factor, and the segmentation and deformation errors also vary between different cases.^1^ However, the correlation and prediction errors that are associated with the accuracy of the correlative and predictive models in XLT system are less susceptible between different cases. In this study, the correlation error is 0.88 ± 0.41 mm, which means that the target with a smaller volume (A_min_ < 35 mm) will be more susceptive to the correlation errors. In the A_min_ < 35 mm scenarios, the percentage of cases with optimized collimator diameters above A_min_ is 40% (Fig. 5). This ratio is much higher than in the A_min_ > 35 mm scenarios. This result suggests that the susceptibility of a target with smaller volume also influences the selection of the collimator diameter. In order to keep target coverage, the larger collimator diameter (Φ > A_min_) was selected using an optimization strategy in small target volume cases.

A considerable amount of work needs to be further improved on CVH and LVH analysis. The CVH/ LVH evaluation needs to be replaced using DVH/normal tissue complication probabilities models in order to evaluate the actual dose delivered to the CTV and surrounding lung tissues. The entire treatment delivery process needs to be taken into account in the temporal interplay between CTV and the temporal aspect of actual dose delivery.^25^, ^26^, ^27^ Additionally, the PTV border volume, which is usually lung tissues rather than tumor tissues, used to compensate for treatment uncertainties, the Monte Calro dose calculation accuracy is needed. In this study, we only considered the treatment correlation and prediction errors, and the target motion is considered rigid. Our subsequent studies will focus on tumor segmentation and deformation errors in the treatment delivery process, and we hope to establish a complex model of the tumor movement. Multiple collimator combinations were selected rather than a single Iris collimator in lung CKS treatment. The different sizes of collimator are able to provide more freedom in the plan optimization and improving plan quality. This collimator selection method can also be used in two (small and large) or more collimator combination CKS plans. The collimators can be initially analyzed independently, and then their weights can be combined to the total plans.

In conclusion, the CVH and LVH analysis provided an a‐posteriori quantitative evaluation of the tradeoff between target coverage and normal tissue sparing. The proposal collimator diameter is 1.1 times that of the short axis length of the target volume in a majority of cases at our institution. This optimization strategy can provide us an evidence to select collimator diameter prior to the CyberKnife XLT plan optimization stage. This collimator selection method is also applicable to fixed collimator and synchrony plans.

## CONFLICT OF INTEREST

The authors declare no conflict of interest.
